# Supplementary figures and images for: GENERALIST: A latent space based generative model for protein sequence families
Source: PLoS Comput Biol. 2023 Nov 27;19(11):e1011655. doi: 10.1371/journal.pcbi.1011655 (PMC10703406; doi:10.1371/journal.pcbi.1011655)

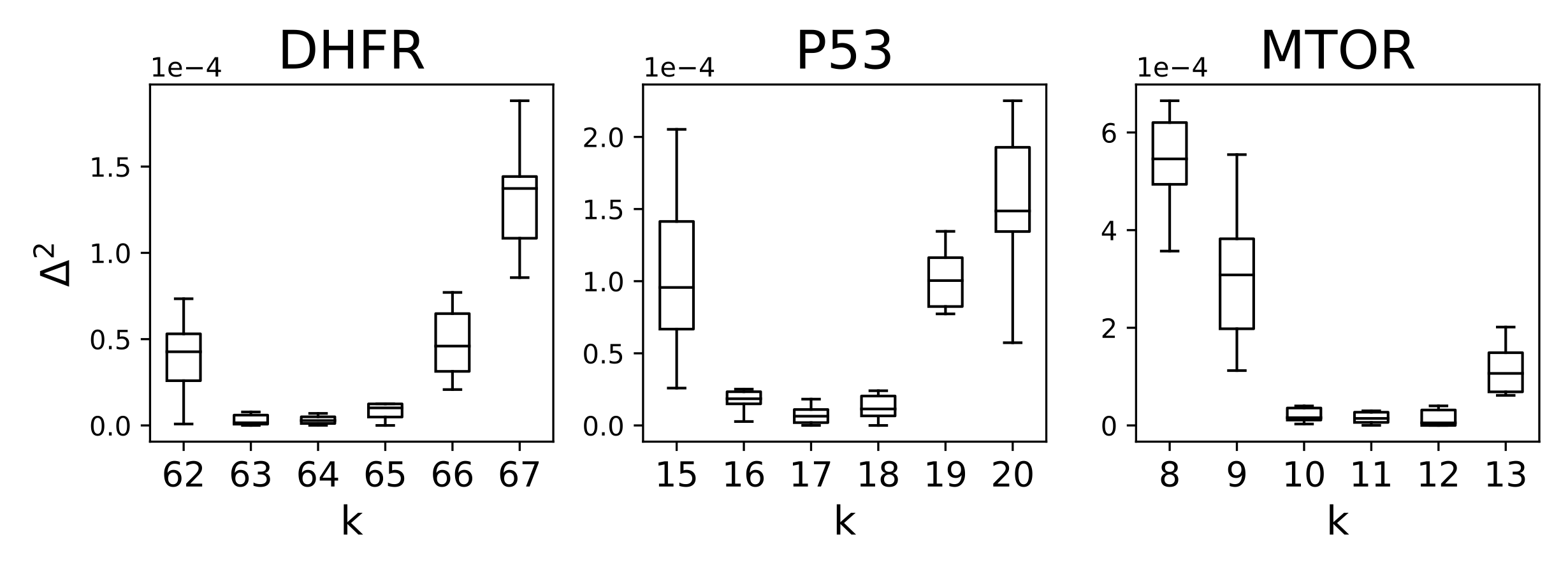

Supplement: S1 Fig — The optimized value is Δ2 = (〈Hmin from generated ensemble to MSA〉−〈Hmin within MSA〉)2. Each box plot represents 10 runs for each latent dimension. The optimum latent dimension for DHFR is 64, for P53 is 17 and for and for MTOR is 12. (TIF) [file pcbi.1011655.s001.tif]

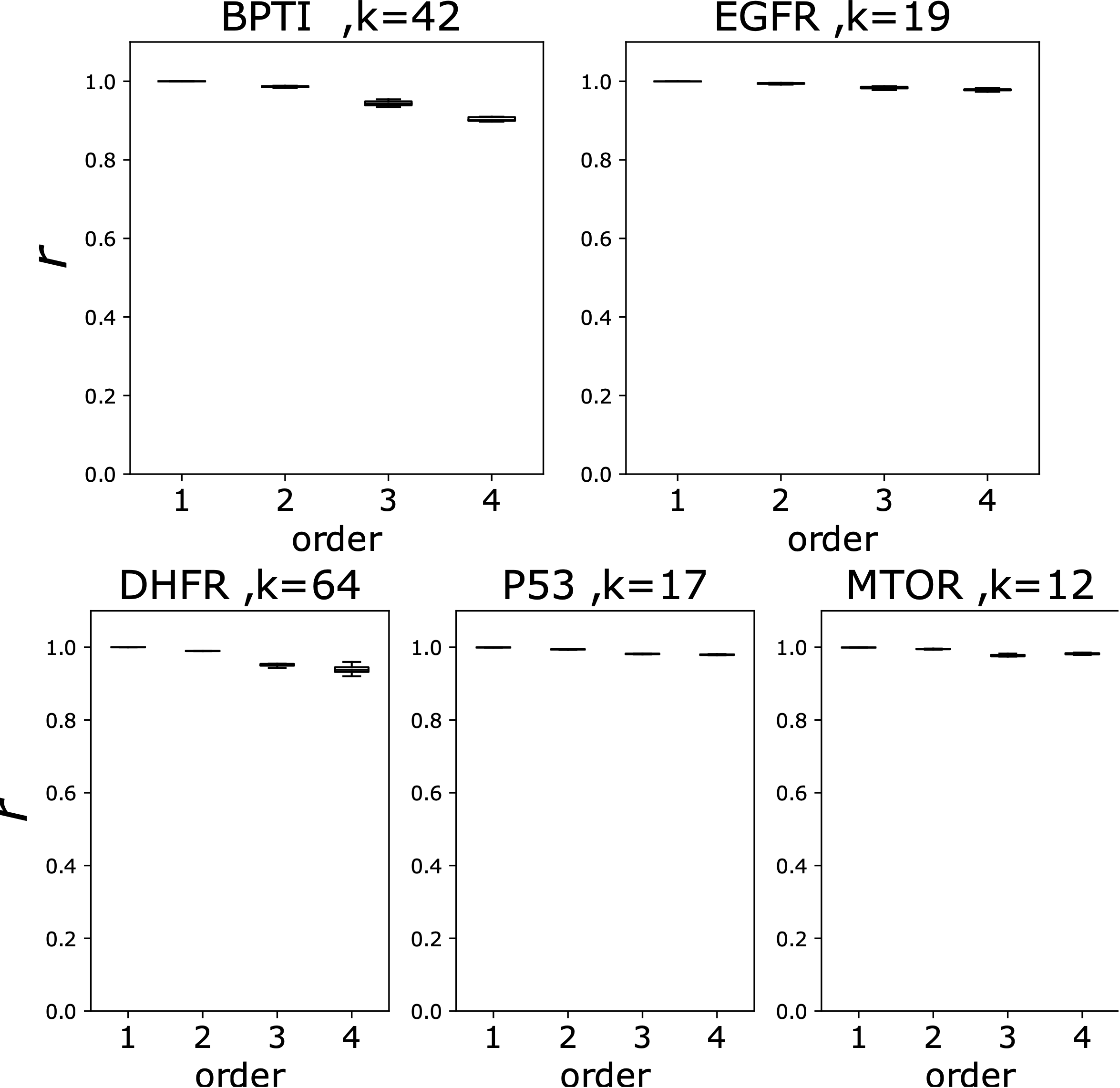

Supplement: S2 Fig — Pearson coefficient of correlation r (y-axis) generated from calculating the cumulants of the generated ensemble and the natural ensemble vs order of cumulants n (x-axis). Each box represents 10 runs for the same latent dimension of GENERALIST. (TIF) [file pcbi.1011655.s002.tif]

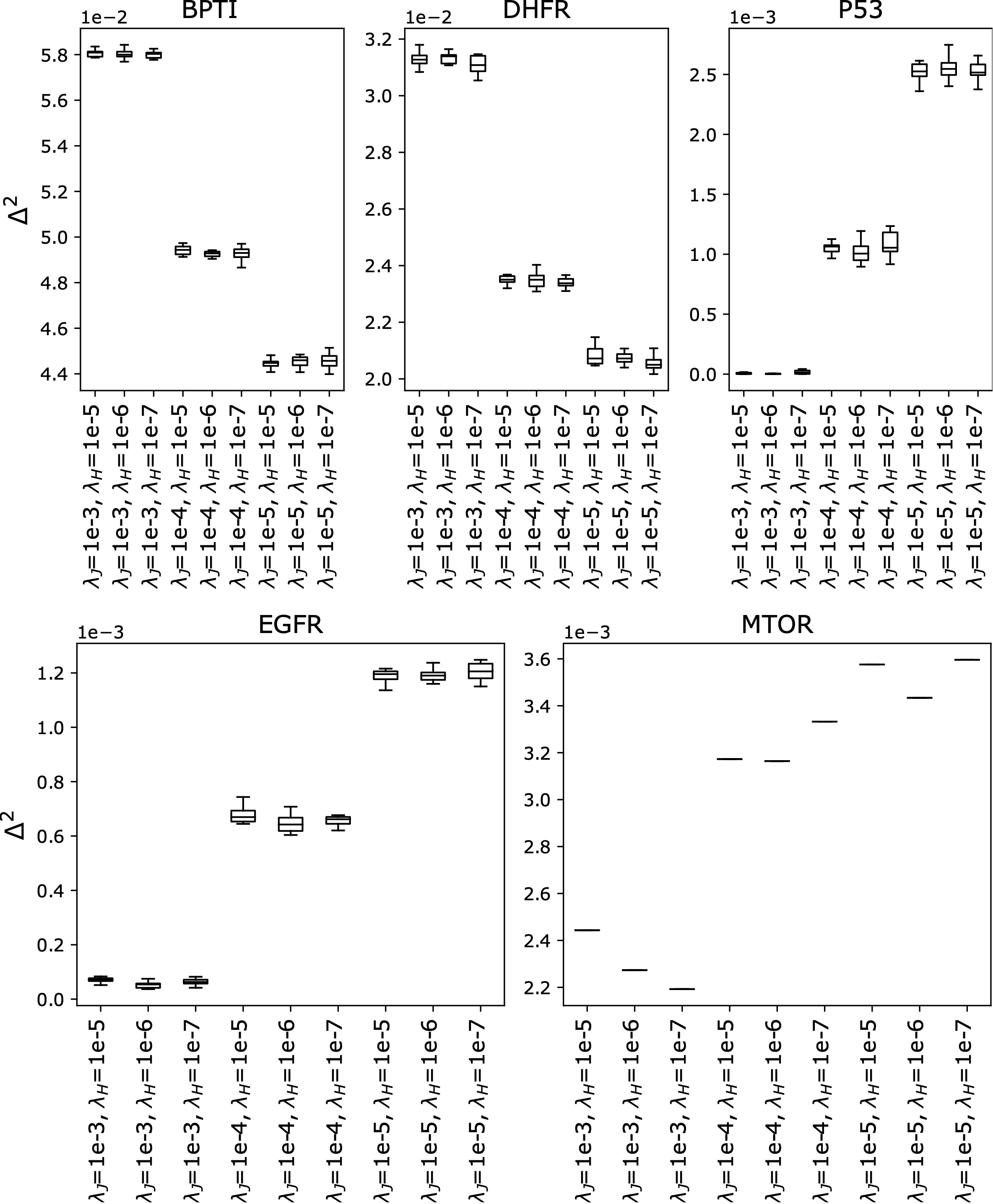

Supplement: S3 Fig — The regularization for the couplings and the field, λJ and λH respectively. The optimized value is Δ2 = (〈Hmin from generated ensemble to MSA〉−〈Hmin within MSA〉)2. (TIF) [file pcbi.1011655.s003.tif]

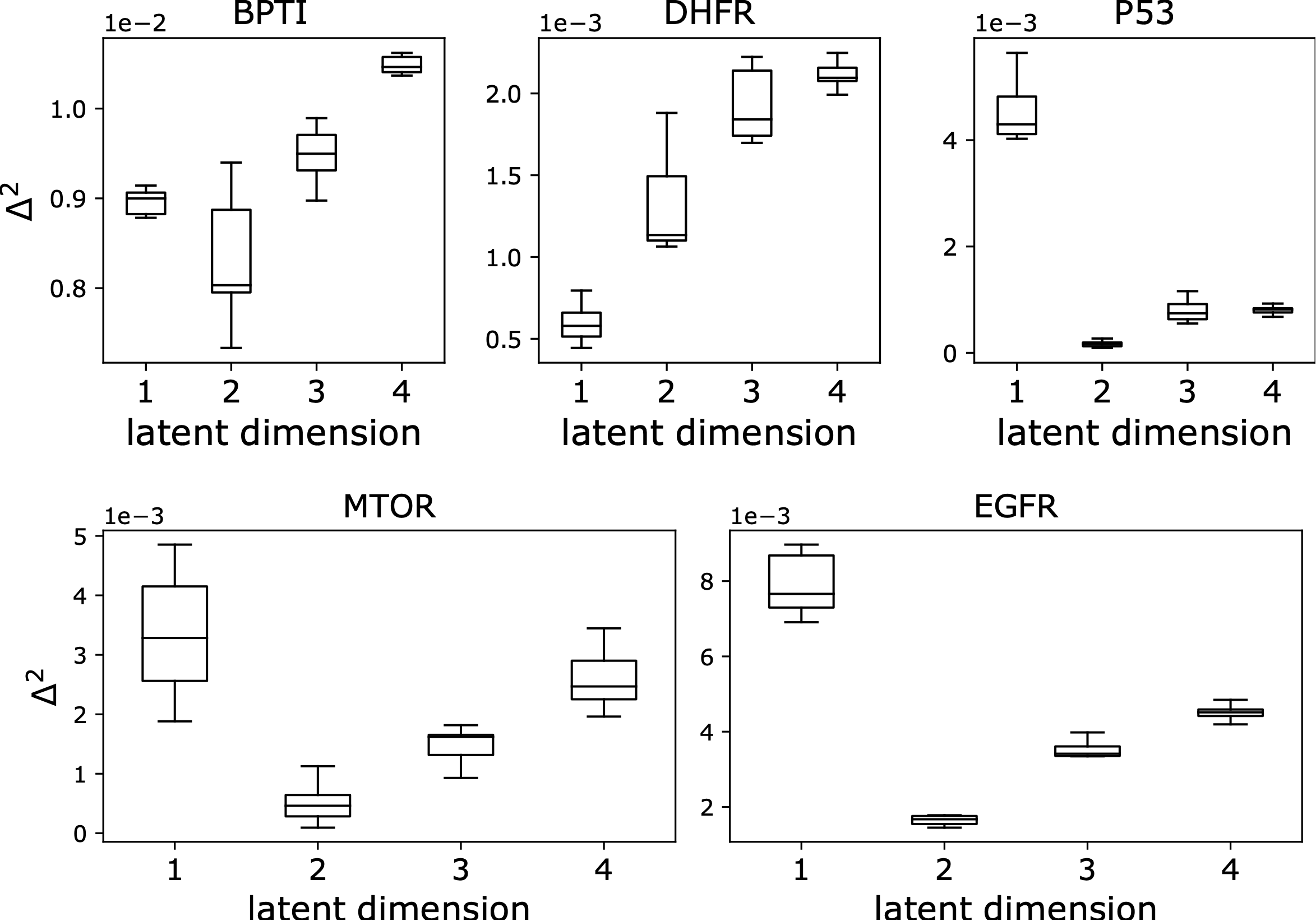

Supplement: S4 Fig — The optimized value is Δ2 = (〈Hmin from generated ensemble to MSA〉−〈Hmin within MSA〉)2. (TIF) [file pcbi.1011655.s004.tif]

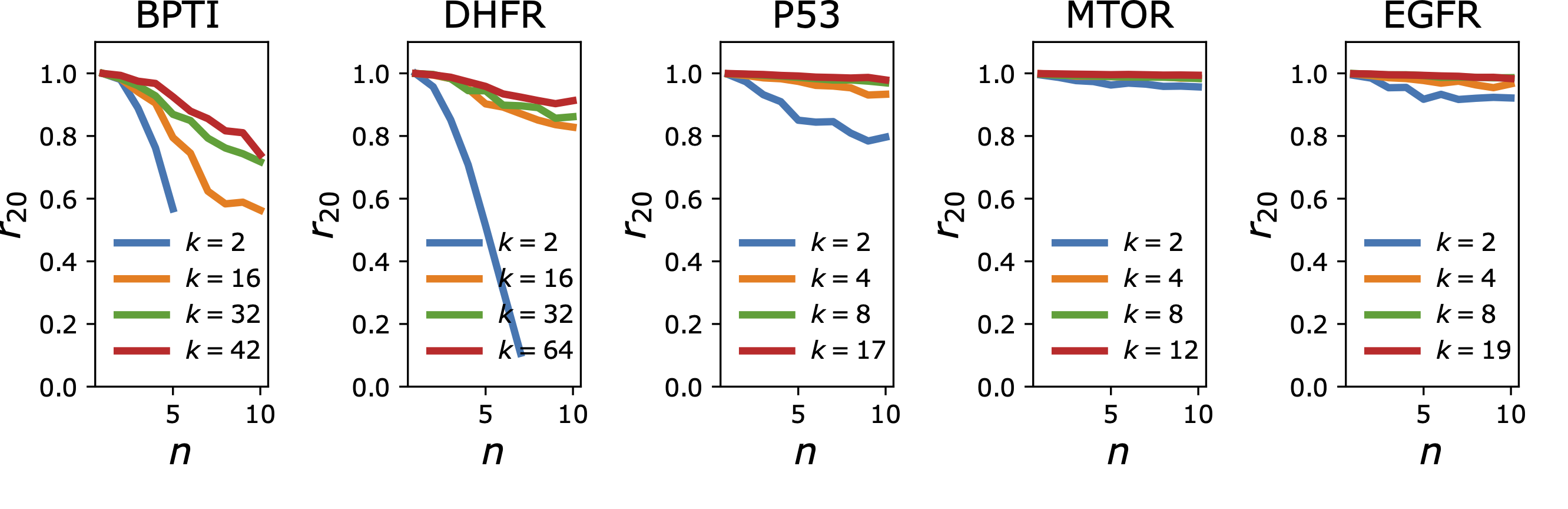

Supplement: S5 Fig — The average Pearson correlation coefficient between frequencies of top 20 amino acid combination of order n (x-axis) averaged across different combinations (y-axis) for GENERALIST model trained with different latent dimensions K (legend). Each subplot represents a different protein from left to right: BPTI, DHFR, P53, EGFR, MTOR. (TIF) [file pcbi.1011655.s005.tif]

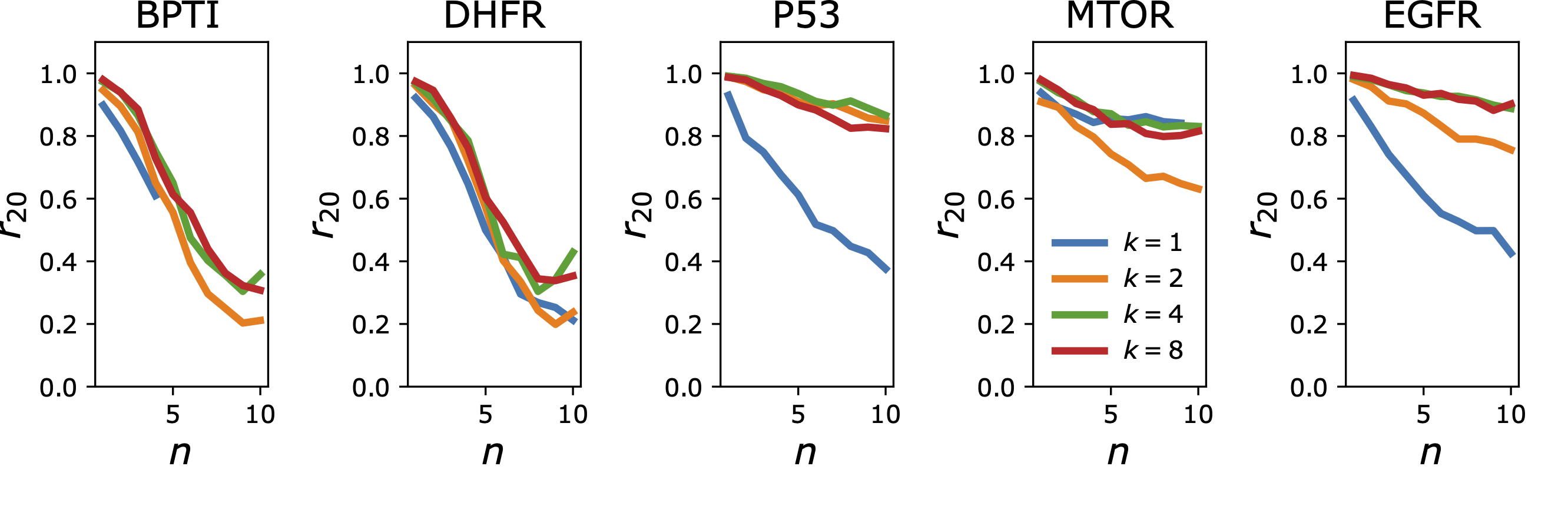

Supplement: S6 Fig — The average Pearson correlation coefficient between frequencies of top 20 amino acid combination of order n (x-axis) averaged across different combinations (y-axis) for VAE model trained with different latent dimensions K (legend). Each subplot represents a different protein from left to right: BPTI, DHFR, P53, EGFR, MTOR. (TIF) [file pcbi.1011655.s006.tif]

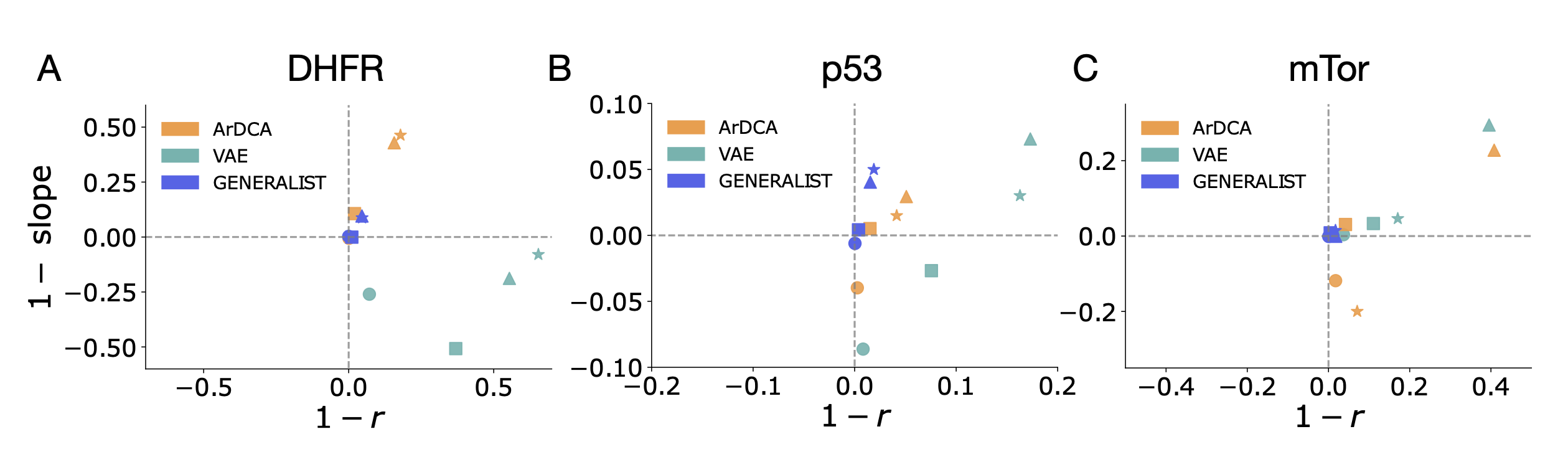

Supplement: S7 Fig — For each order of statistics (grey legend panel A), the frequencies of different amino-acid strings are obtained from Natural and generated ensemble. For order >2, mean removed frequencies are used. The slope of the best fit line of those frequencies as well as the Pearson coefficient of correlation is obtained. 1—slope (y-axis) vs 1—Pearson correlation (x-axis) is plotted for different models (legend). (TIF) [file pcbi.1011655.s007.tif]

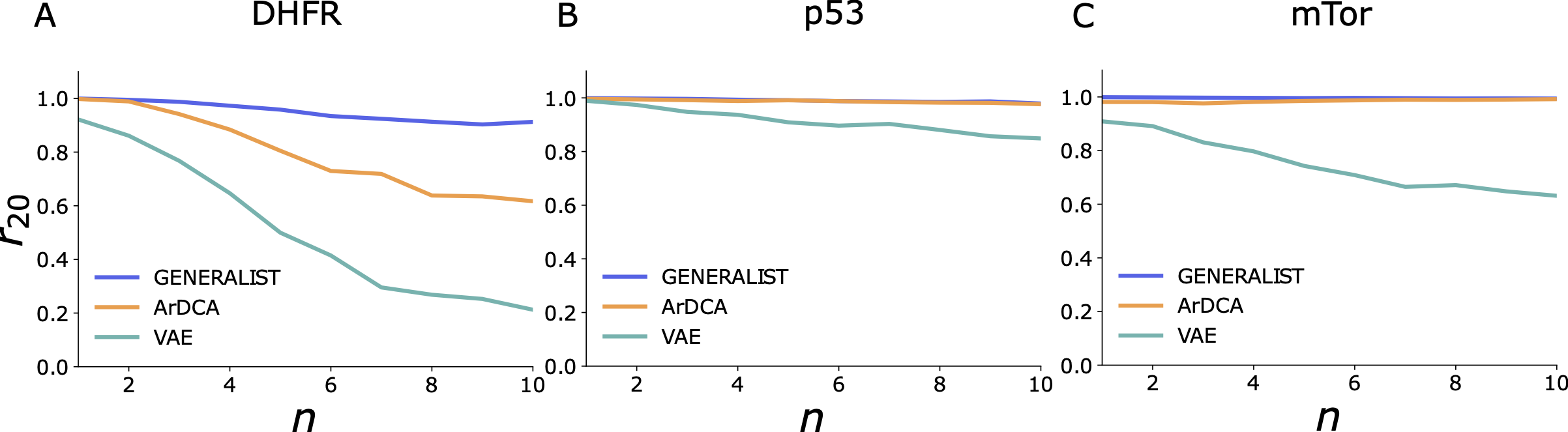

Supplement: S8 Fig — The average Pearson correlation coefficient between frequencies of top 20 amino acid combinations of order n (x-axis) averaged across different combinations (y-axis). Panel A, protein DHFR, panel B protein P53 and panel C is protein MTOR. (TIF) [file pcbi.1011655.s008.tif]

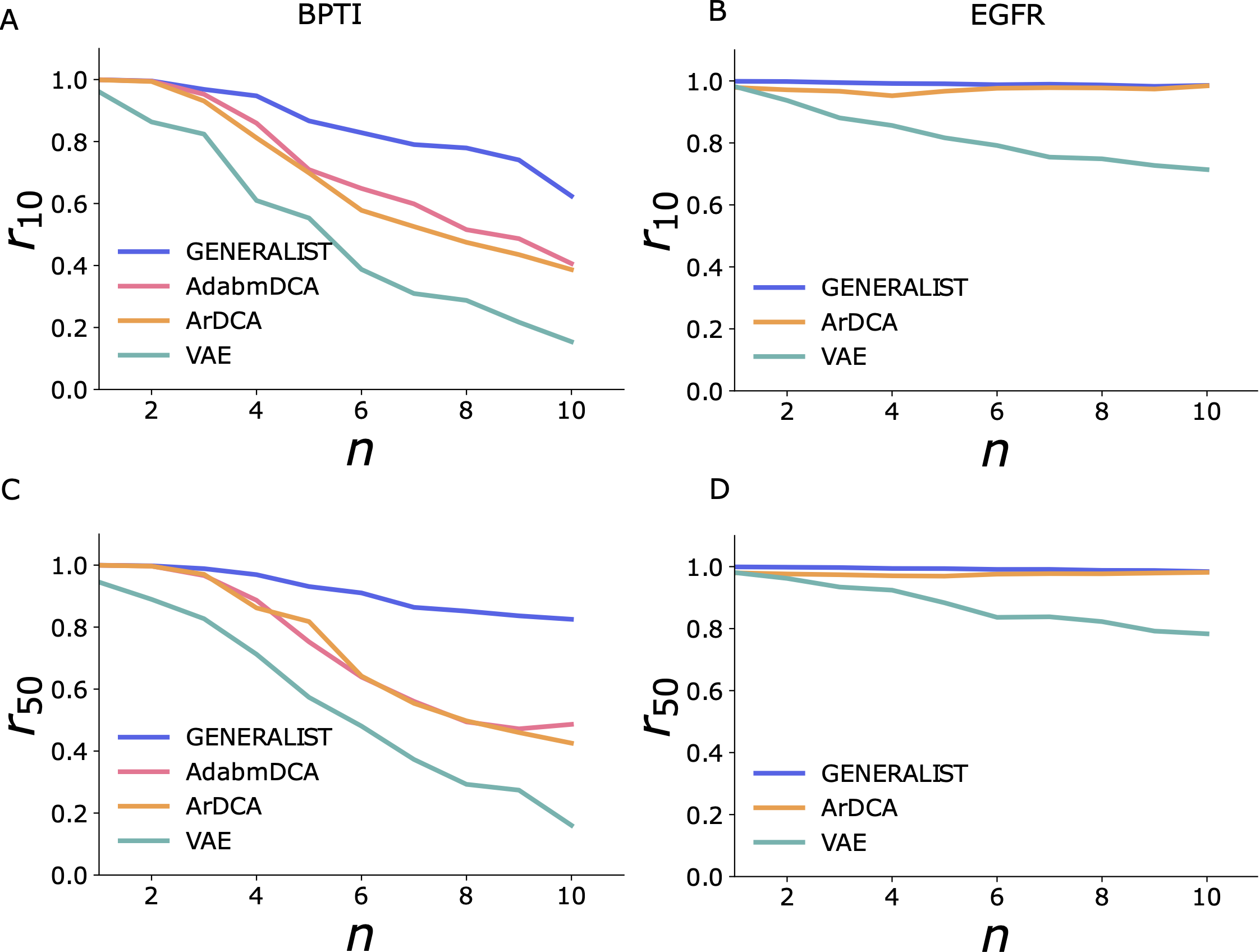

Supplement: S9 Fig — Panels A, B. The average Pearson correlation coefficient between frequencies of the top 10 amino acid combinations of order n (x-axis) averaged across different combinations (y-axis). Panels C, D Same as A and B but using the frequencies of the top 50 amino acid combinations to obtain the average Pearson correlation. (TIF) [file pcbi.1011655.s009.tif]

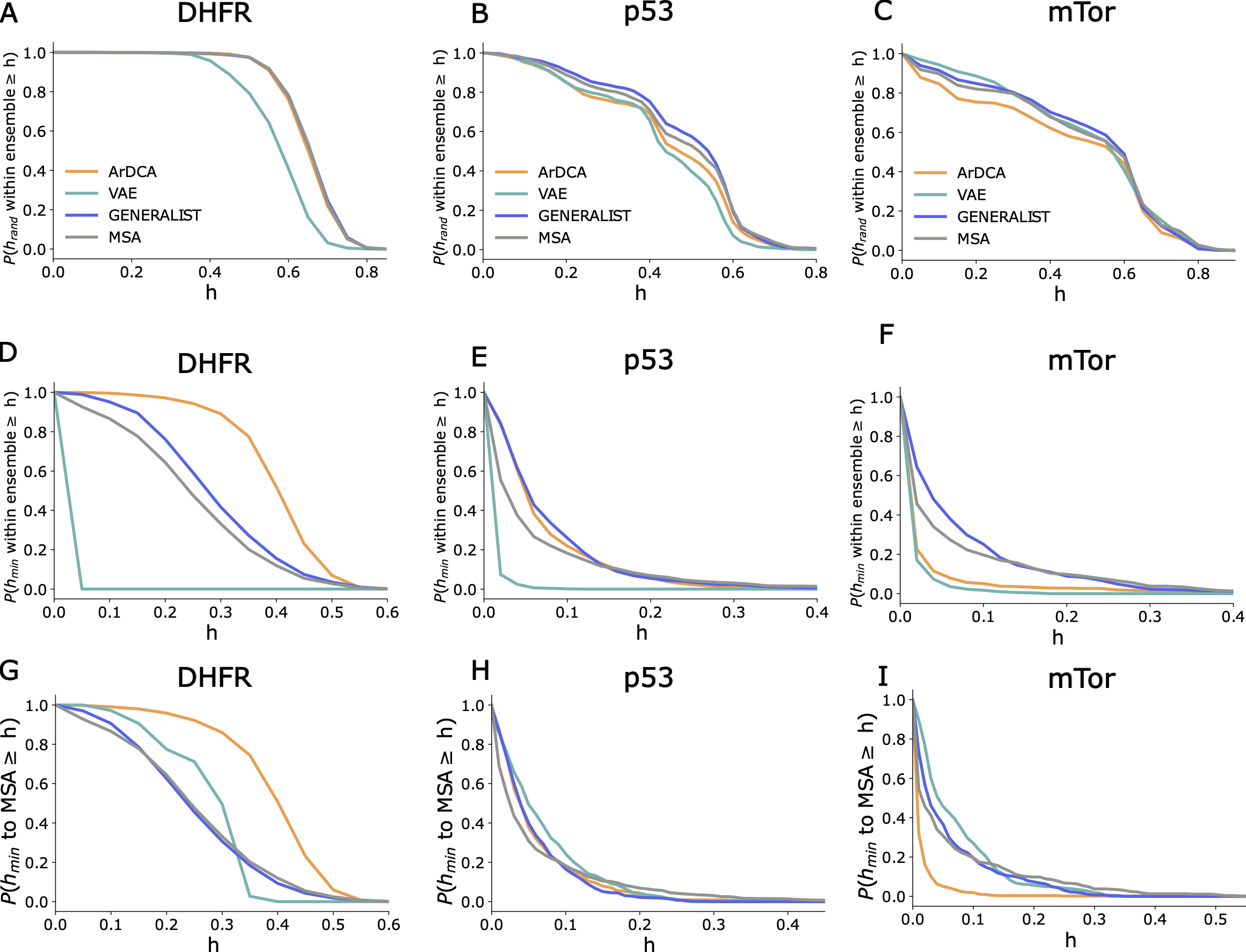

Supplement: S10 Fig — Panels A, B and C. Distribution of fractional Hamming distances between random pairs of sequences within an ensemble shown as the fraction of pairs for which the hamming distance hrand (y-axis) is greater or equal than value h (x-axis). Panels D, E and F. Distribution of fractional Hamming distances to the closest sequence within an ensemble for different models shown as the fraction of sequences for which the minimum hamming distance hmin(y-axis) is greater or equal than value h (x-axis). Panels G, H and I. Distribution of fractional Hamming distances to closest natural sequence for different models shown as the fraction of sequences for which the minimum hamming distance hmin to MSA (y-axis) is greater or equal than value h (x-axis). (TIF) [file pcbi.1011655.s010.tif]

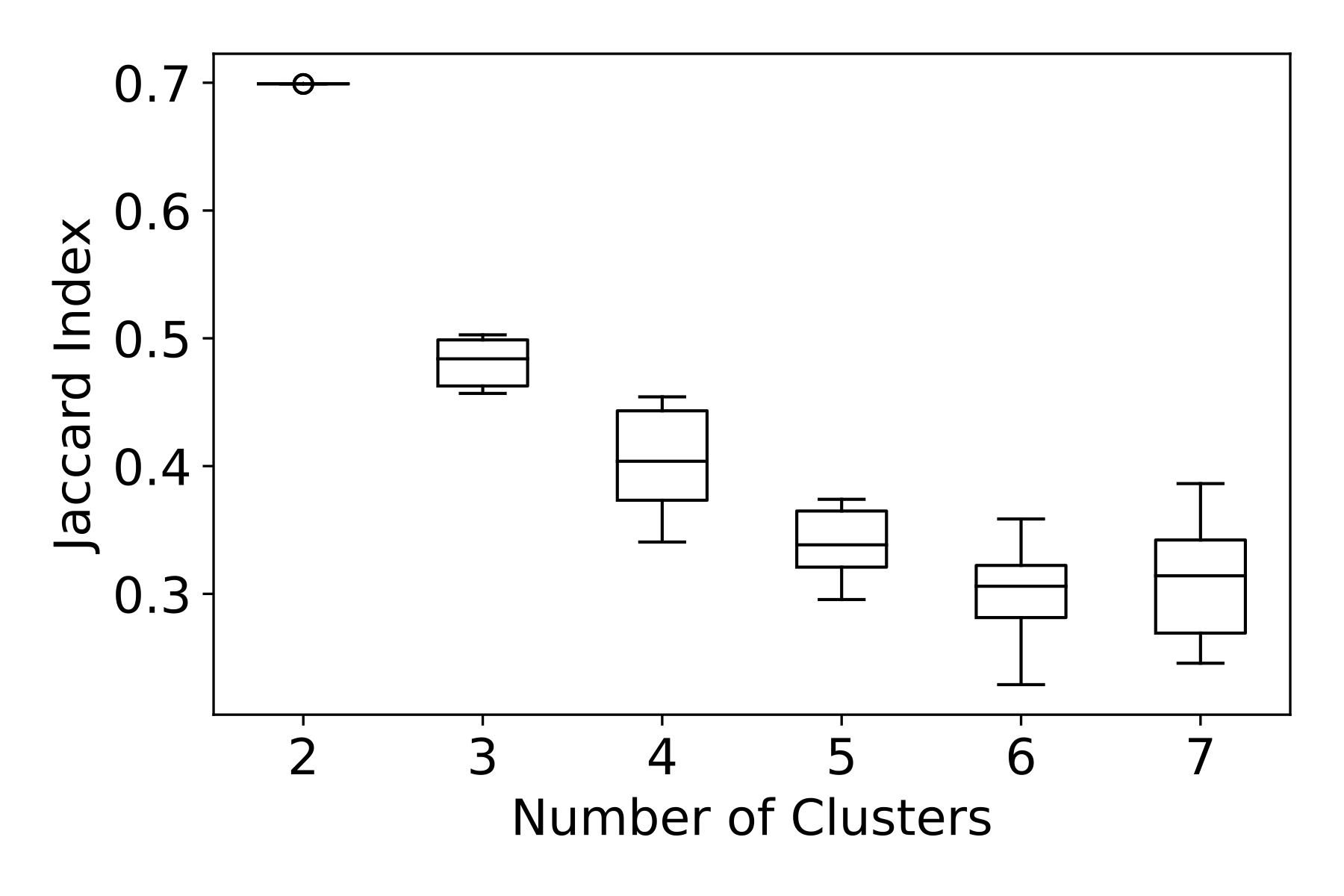

Supplement: S11 Fig — The optimal value is N = 2. This was chosen with the Jaccard Index, a measure of similarity between cluster assignments. Each box plots represents the Jaccard Index over 20 iterations of comparing the assigned clusters of two GMMs for the labeled number of Gaussians. (TIF) [file pcbi.1011655.s011.tif]
