# Supplementary material for: GENERALIST: A latent space based generative model for protein sequence families
Source: PLoS Comput Biol. 2023 Nov 27;19(11):e1011655. doi: 10.1371/journal.pcbi.1011655 (PMC10703406; doi:10.1371/journal.pcbi.1011655)
Supplement: Attachment — Submitted filename: review.pdf [file pcbi.1011655.s013.pdf]

An efficient generative model for protein sequence families

The authors propose a method called *Generalist* to model the sequence variability in a protein family and to generate novel protein sequences. The method is based on the idea of a latent space: a hidden state $\vec{z}_n$ of dimension K is attached to each sequence n in the protein family, and sites are independent for a fixed state of the hidden variables. Authors show that their method generates sequences with statistical properties very close to those of the MSA, accurately reproducing high order statistics. They also show that generated sequences are relatively distant from those found in the MSA, showing the model is able to generalize.
I found the model and results very interesting and the paper relatively clear. However, I found that the authors remain a bit superficial in the analysis of their results, even in the SI. I also have doubts about whether the good performance of *Generalist* in reproducing statistics of sequences is due to it also fitting phylogenetic relations between sequences, which is something I believe a generative model should avoid (or at least it should be discussed).
I list below many comments going more in detail.

Comments

1

If found the paragraph about the mathematical aspect of *Generalist* a bit confusing.

- as far as I understand, π_{anl} is the probability to observe state a at position l *given* a state $\vec{z}_n$ of the latent space. This is a general relation and is unrelated to the training data and the alignment, but this is not clear from the text. Why not use a more explicit notation such as $P(\sigma_{a,l}|\vec{z}_n)$? Maybe this is a misunderstanding on my part.
- I found the sentence "Sequences are modeled as arising from their own Gibbs-Boltzmann distribution" (l. 128) to be a bit confusing. Shouldn't it be something like "Given a state of the latent variables z, a sequence is distributed according to a Gibbs-Boltzmann distribution containing only fields". Of course,, a state of the latent variables is attached to each sequence of the alignment.
- One has to read the SI to understand how sequences are actually sampled. Given that the main point of the article is the statistical properties of sampled sequences, I think this could go into the main text.

2

The authors use Potts models and Variational Auto-encoders as a comparison to *Generalist*. However, it seemed to me that Restricted Boltzmann Machines are a very natural comparison. The main difference between RBMs and *Generalist* is that in the latter, hidden units (or latent variables) are fixed to some predetermined states $\vec{z}_n$, one for each sequence of the original alignment. In both setups,

once the latent state is fixed, all positions in the sequence are chosen independently.
I was a bit surprised to not find any discussion of this connection with RBMs, and am curious to ask the authors what they think about it.

3

A question about training: is the likelihood a convex function? Does it have a clear minimum?

Illustrating this, I ran a simple numerical experiment using the code and notebooks that the authors provided. I trained *generalist* on an alignment of 400 sequences of length 2 being either "AC" or "CA". The magnetizations are such that letters "A" and "C" are heavily favored over other amino-acids, and there is a very strong correlation between the two positions.
I used a latent space dimension $k = 1$. As far as I understand, *generalist* should be able to perfectly reproduce the statistics with parameters such as

- fields $\theta_1 = \theta_0 \cdot [-1, 1, 0, \ldots, 0]$ for the first position and $\theta_2 = \theta_0 \cdot [1, -1, 0, \ldots, 0]$
- "temperatures" $z > 1$ for the "AC" sequences and $z < -1$ for the "CA" sequences.
  At sampling time, if $z > 1$ is chosen, the most likely sequence is "AC". If $z < 1$ is chosen, the most likely sequence is "CA".

Here were the results:

- with the default settings, *generalist* did not converge to the above described state, but instead to a state with $\theta_1 \simeq \theta_2 \simeq [-1, -1, 1, \ldots, 1]$ and $z \simeq 2$ for all sequences. Of course, this reproduces the magnetizations, but not the correlations, as the four states "AA", "AC", "CA", "CC" will have the same energy.
- when I initialized the model with the fields $\theta_1 = \theta_0 \cdot [-1, 1, 0, \ldots, 0]$ and $\theta_2 = \theta_0 \cdot [1, -1, 0, \ldots, 0]$, training nicely converged to the solution proposed above.

I conclude from this that training highly depends on the initial state of parameters, and that the likelihood may posess several local maxima. However, I could see no mention of this in the article. Did I misunderstand something?
I think the authors should comment a bit more on the training procedure: does it strongly depend on the initial conditions? does the optimal k vary from training to training? why are the z initialized to be positive values, whereas using negative values could increase the expressiveness of the model?

4

The authors find optimal values for the dimension K of the latent space using a quite interesting method, avoiding overfitting. I have questions regarding the results.

- The optimal K varies significantly for different families: from $K = 10$ for mTOR to $K = 70$ for DHFR. I would appreciate a comment of the authors on this variation. Was that expected from the diversity of sequences found in the different alignments? Looking at Figure 2, it seems that the least diverse alignments have the lowest K: this could easily be explored quantitatively.

- How robust is the optimal value of K? For instance for BPT1, going from $K = 42$ to $K = 50$ completely changes the distribution of Hamming distances. How much do these values change, say, when using different initial conditions for the training? As a suggestion, maybe plotting the optimized quantity as a function of K would give the reader more intuition?
- It would be interesting to see how the reproduction of 2nd and higher order statistics behaves as a function of K. Curiously, this is done for VAE in the SI, but not for *Generalist*.

5

In figure 5, the authors show that for large alignments, the minimal hamming distance between pairs of *Generalist* generated sequences and the minimal distance to a natural sequence is close to the ones found for the natural MSA and on average much lower than when using VAE and Potts models.
First, this result is not completely surprising as K has been optimized exactly for this. Seconds, I have the impression that this means *Generalist* reproduces the phylogenetic bias found in the natural sequences. If this is the case, the good performance of *Generalist* in reproducing high order statistics could be due to the fact that these statistics are indicative of phylogenetic relations and not of functional constraints. These are relations that Potts models try *not* to learn. I think it is important that the authors this potential pitfall.

6

The authors use Potts models as a comparison for *Generalist* and discuss them at length in the introduction. A major difference between Potts models (and also RBMs) and more involved techniques such as VAEs or neural networks is that the parameters of the former have a biological interpretation (to some extent). This is even pointed out in the introduction, as a downside of deep generative models (l. 90).
Did the authors explore possibilities to interpret the inferred parameters of *Generalist*?

Minor comments

- In the introduction (around l.78), the authors make well-argued case against hyper-parameters used in training Potts models. Does *Generalist* use regularization? If not, how does one avoid inifinite values for some parameters? The optimizer ADAM also has some hyperparameters: do those influence predictions (if the likelihood has a single minimum, I imagine they should not)? I think the article would benefit from a more explicit discussion of these issues.
- In the paragraph starting at line 140, the authors describe the protein families used in the article. I am a bit annoyed by the use of the "approximate" sign $\sim$. Why not give the exact values of number of sequences and amino acid lengths? Also, a table would be really helpful here: it could contain number of sequences, length, optimal K and fitting quality for the different families.
- Code: it would be nice to provide installation instructions. Generalist depends on many other python libraries (torch, numpy, etc...), and I had to "manually" figure out which and install them.
- l.73 - "inefficient" might not be the best word. Maybe "computationally hard"?
- l.308 - I am not sure that one can refer to Potts models as "physics-based".
