# Supplementary material for: GENERALIST: A latent space based generative model for protein sequence families
Source: PLoS Comput Biol. 2023 Nov 27;19(11):e1011655. doi: 10.1371/journal.pcbi.1011655 (PMC10703406; doi:10.1371/journal.pcbi.1011655)
Supplement: Attachment — Submitted filename: response_to_reviewers_final.docx [file pcbi.1011655.s014.docx]

Dear Editors and Reviewers

We sincerely thank the three reviewers for their detailed comments on the manuscript. We also thank the editors for giving us an extension to finish our comprehensive analysis. We have now completed our analyses and significantly rewritten the manuscript. We hope that the changes in the manuscript satisfy the reviewers’ concerns.

Salient changes are as follows:

1. We have moderated our claims about our approach and clarified in appropriate locations the details of the calculations. We have also clarified the language as suggested by the reviewers.
2. We have now reformulated our learning procedure following the suggestions from the reviewers. This was to ensure that our models were not stuck in a local optimum. We have also optimized the learning procedure of other models. We have now rerun all our analysis with the improved model fits.
3. We have added an extra section on the value of learning a latent space representation of the sequences.

These changes have made the manuscript much more readable and the presented results much more robust. Below, we attach our point-by-point response.

Best

Purushottam Dixit

Reviewer's Responses to Questions

**Comments to the Authors:**

**Please note here if the review is uploaded as an attachment.**

**Reviewer #1**: In this paper, Akl and collaborators describe a new generative model framework for describing properties of protein families. Their approach uses a set of latent variables, the number of which is chosen by comparisons between the statistics of the models that they infer and the real data, to quantify the probability for particular sequences. They apply their method to data sets for several proteins. Here they show that their method is able to reproduce statistics of the data that in some ways appears to exceed other, alternative approaches.

The method used by this paper is novel, and new contributions in this area are welcome. However, the ideas presented here are in some cases poorly explored. Some characterizations of past work also seem incomplete. In addition, I have technical questions about the work. My detailed comments are below:

1. Justification for the new approach. In multiple places, the authors attempt to justify their approach with arguments about computational efficiency or scaling. For example, lines 72-74, referring to Potts models: “The associated numerical inference is computationally inefficient, limiting their application to small proteins and protein domains ( ∼ 100 residues).” There are also claims about large numbers of hyperparameters for Potts models. There are several problems with these justifications for the current work.

First, the claims above are not generically true. Potts models have been trained using a variety of algorithms for large proteins. This includes work by (Hopf et al, Nat Biotechnol 2017) using pseudolikelihood, or (Louie et al, PNAS 2018) using a method known as minimum probability flow, among others.

We thank the reviewer for this comment and apologize for the confusion in our claims. Indeed, several computationally efficient approaches have been devised to learn Potts models including those referenced by the reviewer. However, while these approximate methods can potentially predict the effect of mutations and physical contacts between residues, they perform poorly when it comes to reproducing statistics of real sequences, which was the chief goal of our generative models and also of our work. The inaccuracy of approximate inference methods (pseudolikelihood, mean field, adaptive cluster expansion, etc.) in reproducing statistics of natural sequences was extensively explored by Figliuzzi et al. [1]. We have now changed our text to reflect this subtle point (lines 76-78).

Indeed, the authors themselves use other algorithms for inferring Potts models in this very paper on proteins with L > 2000.

We apologize for our confusing language. We use the term “Potts models” to refer to a generalization of spin-glass models for categorical data wherein the “Hamiltonian” of any sequence is given by

$$H\left( \boldsymbol{\sigma} \right)=\sum_{i,a} h_{ia}\sigma_{ia}+\sum_{i,a,j,b} J_{iajb}\sigma_{ia}\sigma_{jb}. (R1)$$

In Eq. R1, $"i"$ and $"j"$ represent amino acid positions and $"a"$ and $"b"$ represent protein residues. $"h"$ and $"J"$ are model parameters that are typically inferred from data.

We note that in our manuscript, we do not train Potts models for proteins with L > 2000. In fact, we only train the Potts model for BPT1 with L ~ 60 residues. What the reviewer may be referring to is our implementation of a previously developed autoregressive model (ArDCA) [2] which has a very similar probability distribution as the Potts model yet cannot be represented using a Hamiltonian in Eq. R1. A significant conceptual departure between the two models as noted in the original work that introduced the ArDCA model is that the quantities $"J"$ that couple different positions and amino acids with each other are symmetric in the Potts model but not necessarily so in the autoregressive model. In the attached supplementary note for reviewers, we show the difference between the two models.

While some methods may employ multiple hyperparameters, most really only use a couple of regularization strengths (one for “fields” and one for “couplings”) and perhaps a parameter for data compression. This is not very different from the authors’ proposed method, where the number of latent variables is unknown and must be estimated.

We thank the reviewer for the comment. However, compared to MCMC-based training methods, our GENERALIST approach indeed has a smaller number of hyperparameters that cannot be determined from the data. We do not include the dimension of the latent space as a hyperparameter since its value can be computed from the data. Specifically, the Potts model approach (adabmDCA) requires specification of several hyperparameters not only for regularization but also for collecting statistics from the MCMC chains (number of chains, length of chains, sampling times, wait times, and the proposal distribution). The performance of MCMC methods can depend heavily on these hyperparameters [3]. In comparison, GENERALIST requires a maximum of 5 hyperparameters. These include gradient stopping threshold, step size, and 3 parameters if adam optimization is used.

Second, despite the claim that computational efficiency is important, the paper never presents any comparison of run time or other computational parameters for the various methods that they test. This is surprising, and it makes claims about “efficiency” impossible to evaluate in a quantitative way.

We agree with the reviewer that we do not support our claims of computational efficiency. We have now removed these claims from the manuscript.

Finally, and perhaps most importantly, the justification of computational efficiency doesn’t seem necessary at all. Why not simply motivate this work by emphasizing the latent variable approach, which differs from most methods in this area, and the statistical properties of the model?

We thank the reviewer for this suggestion. We have now included additional analyses that show how the latent space representation can be used to identify clusters of sequences that have differing covariance structure (lines 345-367).

2. Quantitative comparisons with other methods. In Figure 3, if my understanding of the SI is correct, then the higher order correlations presented are not actually connected correlations (except for the second order ones). Instead, the expected “mean,” given by the product of individual AA frequencies, is subtracted from the frequency in simulations. In this case, the “higher order” terms can actually depend substantially on lower order moments. To truly get independent statistics, one should instead use the connected correlations or cumulants.

We thank the reviewer for the comment and apologize for our confusing presentation. In our analyses (Figure 3), we indeed use cumulants rather than simply removing mean values from higher order correlations. That is, we first remove the mean by calculating $\delta\sigma_{i}= \sigma_{i}-\langle\sigma_{i}\rangle$. And then obtain the product across different residues/amino acid positions to obtain the cumulants. These cumulants are identical to the connected correlations up to order 3 and small differences emerge only from order 4 onwards. Notably, we only report our results up to order 4. We have changed the manuscript to describe our calculation as cumulants instead of “mean-removed statistics” to avoid confusion (lines 207-208).

More generally, for statistics that involve large numbers of sites/residues, it would be helpful for the authors to justify why one should believe that these statistics are meaningful in the face of finite sampling noise. Surely, correlations for combinations of ~ 10 residues will be woefully undersampled in any data set of realistic size, for example. Simulations could help the authors to explore these issues.

The reviewer’s concern is indeed well-founded. To avoid under-sampling, the metric $r_{20}$ (which was previously established by the group of Ron Levy) [4] computes the correlations only among top 20 most common occurrences combinations. The original manuscript has established the metric and its properties in detail and has studied it extensively. That study included investigations of simulations of real and *in silico* datasets to ensure stability and robustness to noise. Moreover, in our work, we also compute related metrics $r_{10}$ and $r_{50}$ (SI Figure 11) to avoid biases related to choosing only 20 common amino acid combinations. We have now clarified this in the manuscript (lines 220-221).

In Figure 4, the authors compare several aggregate statistics for different models. Figures 4A-B seem to suggest that sequences from GENERALIST are typically highly similar to ones from the MSA and inferred “optimal” sequences. Is it clear that GENERALIST is significantly exploring the landscape of possible sequences?

We thank the reviewer for the comment. In Figures 2, 3, and 5 we extensively investigate the sequences sampled by the generative models for both accuracy and novelty. Analysis in Figures 3 and 5 shows that GENERALIST can explore the sequence landscape quite well and can innovate new sequences while capturing the statistics of natural ones.

In Figure 4, we instead investigate “local optima” of the sequence landscapes, that is, the sequences that correspond to the local maximum of the probability. We stress that these sequences *are not* produced through sampling from a distribution but rather are obtained using a local optimization approach. These calculations are detailed in SI section 6. We have also clarified this point in the manuscript (lines 263-265).

GENERALIST and ArDCA are maximum likelihood models. That is, they are fit to assign highest probability to the observed data. In contrast, adabmDCA (the Potts model) is fit to reproduce data statistics and not to individual sequences. Therefore, it is likely that adabmDCA predicts spurious optima (a well-established concept in Potts models [5]) in the sequence landscape. As we show in Figure 4, the optimal sequences predicted by GENERALIST and ArDCA are likely to fold in stable 3D structures while those predicted by adabmDCA are not.

In addition, it would be helpful for the authors to more thoroughly explain the plddt score, how this is calculated, and the motivation behind its use. How is a plddt score calculated for a particular sequence (natural or artificial)? In principle, sequences with some disorder could also be functional. Is it clear that larger plddt scores are better?

We thank the reviewer for the comment and apologize for the lack of clarity in our discussion. The Local Distance Difference Test (LDDT) [6] is a metric that allows us to analyze how accurate is a model of a protein structure with respect to the experimentally determined structure.

AlphaFold [7] is a novel technology that has solved the 50-year grand challenge of protein structure prediction. It uses AI to make the predictions, and it was trained based on the accumulated knowledge in the protein data bank. During training, the AI not only aims to make   accurate predictions but also quantifies how confident it is in the predicted structure. AlphaFold returns a measure they developed called the predicted LDDT score (plddt). This score is a complex function of the AlphaFold internal computations and cannot be expressed as a simple calculation. Higher values are known to imply a lower uncertainty in the predicted structure. Ranges between 70 and 100 typically yield accurate predictions, while lower values typically correspond to structures that have some discrepancies with the experimental structure [8].

Notably, the reviewer is correct in pointing out that disordered regions that may correspond to low plddt scores may in fact be functional. To avoid this complication, we performed our structural analyses on a protein (BPT1 domain) that in its native state wild type sequence has negligible disorder. Therefore, it is reasonable to expect that functional variants of BPT1 are also likely to have negligible disorder. However, the reviewer has raised an important issue about functionality of disordered regions, and we have now incorporated this concern in the manuscript (lines 292-296).

While statistics of the sequence ensemble are interesting, these may be primarily of specialized interest. In order to make a full comparison with alternative methods, it would be useful to explore how well GENERALIST is able to predict the functional effects of mutations, for example.

We thank the reviewer for the comment. We agree with the reviewer that broadening the scope of GENERALIST to other functionalities is indeed desirable. We believe that reproducing statistics of the training data is the centrally most important characteristic of generative models. Other functionalities, for example, predicting fitness effects are only an indirect evaluation, given that these models are neither trained on them nor are meant to predict them.

More importantly, the current formulation of GENERALIST assigns a specific latent variable for each sequence in the data. When the latent variables are fixed, there are no “interactions” among different amino acid positions. Therefore, GENERALIST in its current formulation does the effect of genetic background (probability of amino acid switch depends on latent space location) but does not have any epistasis (probability of two amino acid changes is the product of the probabilities of single amino acid changes). One way to incorporate epistasis is to learn a distribution $p\left( z \right)$ over the latent space. This requires a significant extension of our current framework using variational inference. We leave these explorations for future work.

Some analyses suggest overfitting of Potts models to particular data sets, but I would be inclined to argue that this is not an entirely fair comparison. The authors fine-tune their own analyses, selecting a number of latent factors that is appropriate for each data set. In contrast, default parameters are used for the different DCA model-fitting methods. One might expect that overfitting could easily be countered by appropriately adjusting regularization in the Potts model methods.

We thank the reviewer for the excellent point. Following the reviewer’s suggestions, we have now performed extensive scan of hyperparameters for the other models, ArDCA and VAE, (Regularization, latent dimension, etc.) to mimic our training of the GENERALIST model (lines 193-196). These results are now presented in Figures S3 and Figure S6. We note that we obtained optimal hyperparameters for adabmDCA (Potts model) through a personal communication with the authors of that paper [3] and therefore did not further optimize its performance. We have now reworded our results in the main text (lines 197-200).

3. Unique contributions of this approach. The authors have developed a detailed method to infer latent factors describing ensembles of protein sequences. Remarkably, however, the potential insights from the latent factors themselves appear to be completely unexplored. Do these factors have any clear biological interpretation? Could they be used for categorizing sequences in any way? Or are these latent factors purely for computational expedience?

We thank the reviewer for this excellent suggestion. Indeed, unlike other methods, the latent space embedding allows us to classify sequences with similar covariance structure, therefore, naturally identifying sectors in the data.

To show this, we used Gaussian mixture modeling (GMM) to cluster latent variable representations of sequences. GMMs imagine that the data (in our case, the latent space descriptors) arise from a mixture of several multivariate Gaussian variables. The parameters of these Gaussian distributions (mean value and covariances) as well as the membership of individual data points to the Gaussians can be determined using well-established algorithms such as the expectation-maximization algorithm. We use a built-in package in python to carry out these calculations.

The optimal number of clusters can in principle be determined using information theoretic criteria. However, given that the data are correlated with each other through an evolutionary process, we used the adjusted Rand index (ARI) which measures the reproducibility of the model [9]. Briefly, the cluster number N with the largest ARI corresponds to the most stable cluster assignment.

As shown in Reviewer Figure 1 (Figure 6), our findings suggest that the clusters generated by this process effectively encapsulate distinct statistical characteristics of the sequences in the training data. Our conclusions stem from an extensive analysis of these clusters, which involved computing the covariance and mutual information within each cluster. Briefly, our analysis identified 4 clusters in the data. The largest of these clusters comprised 73% of the total data and the other 3 clusters comprised 11%, 10%, and 6% of the data. respectively. Unsurprisingly, the largest cluster primarily preserved the statistical characteristics of the entire MSA. However, our investigation into the smaller clusters yielded a variety of results.

For example, as seen in Reviewer Figure 1, we discovered that each cluster conserved different pairs of amino acids, indicating the uniqueness of each cluster. Further, the mutual information between pairs of positions also varied from cluster to cluster. To ensure that these variations were not merely a result of differing cluster sizes, we constructed a null model. The null model involved random assignment of sequences to clusters that mirrored the size distribution of clusters inferred by GMM. Interestingly, these null clusters reproduced the original MSA's statistics much more closely than their GMM counterparts (SI Figure 5).

Reviewer Figure 1. Statistics of clusters from GMM for BPT1. Panel A. Comparison of covariance calculated from natural sequences within each cluster (labeled 0, 1, 2, and 3) and the entire MSA. B. Comparison of mutual information calculated between pairs of positions from natural sequences within each cluster and the entire MSA. Cluster sizes were 1034, 1623, 1774, and 12138 for Clusters 0, 1, 2, and 3 respectively.

4. Model fitting. It would be helpful for the authors to more clearly explain the methods that they have used for fitting their model. Based on eq. (2), one must compute the partition function (or complicated averages) to fully evaluate the derivative. In particular, the pi functions appear to be probabilities within the model rather than in the data, so it’s not obvious how to obtain these without either estimating them through simulation or computing the partition function.

We apologize for the unclear writing. In our model, when the latent variables are specified, the partition function for a sequence is a product of independent partition functions, one for each position. Therefore, it can be evaluated quickly by explicit summation over 21 states. This in fact is the chief numerical advantage of GENERALIST. We have modified the manuscript to explicitly state this advantage (lines 144-147).

Optimization with latent variables is generally non-convex, making this problem complex. How sensitive are the results to initial conditions or any approaches for regularization? Are latent factors similar between runs, or do they appear to be different each time? How does general performance vary between instances?

The reviewer raises an excellent point. Parameter inference in GENERALIST is indeed nonconvex with potential to get stuck in local optima. Based on the comments of this reviewer and others, we now alleviate this issue by training the GENERALIST model multiple times and choosing the one that has the highest likelihood. The latent factors are not similar between different runs, due to a variety of previously documented indeterminacies [10,11]. We now describe this in detail in the manuscript as well as the supplementary materials (lines 191-200).

Finally, sampling from the model could be more completely explained. Once the z’s have been learned, how does one resample the z’s? Are these assumed to come from a distribution?

We apologize for the lack of details. In the current formulation of GENERALIST, the latent variables are assigned as maximum likelihood estimates for each sequence in the MSA. To sample sequences, we sample the learnt z’s with replacement and then sample the sequence using probabilities calculated using Eq. 1 of the main text. We now discuss this in the manuscript (lines 151-155).

5. References. There are several places where the authors could augment or correct references in the current version of the text. In reference to the effects of mutations on fitness, (Ferguson et al, Immunity 2013) precedes ref. 8. (Figliuzzi et al, Mol Biol Evol 2015) extended this concept to protein families. And of course, related ideas have now been applied in many subsequent papers. In line 89, it may be best to refer to (Riesselman et al, Nat Methods 2018) and subsequent work. Regarding the identification of “optimal sequences,” to my knowledge this was first performed in (Barton et al, PNAS 2015), which also provided a biological interpretation for the optimal sequences.

More generally, this field has greatly expanded and deepened in the last ~10 years. It would be helpful for the authors to expand the present references to highlight original research in addition to recent reviews and to point to additional relevant research in this area.

We have implemented the reviewer’s reference suggestion and augmented the reference list.

Minor comments:

The notation for indices used here is a little bit confusing. What is the difference between the position in the protein sequence “l” and the index “n”? Without further explanation it seems like these two coordinates are indexing the same thing, and it’s unclear why both are needed. This is made more clear in the SI, but this should be clarified in the main text.

We apologize for the confusion. The index “n” refers to the sequence in the original MSA, the index “l” refers to the position. Thanks for the suggestion, we have added an explanation of the indices in the main text (lines 141-142).

**Reviewer 2**

The authors propose a method called *Generalist* to model the sequence variability in a protein family and to generate novel protein sequences. The method is based on the idea of a latent space: a hidden state *zn* of dimension *K* is attached to each sequence *n* in the protein family, and sites are independent for a fixed state of the hidden variables. Authors show that their method generates sequences with statistical properties very close to those of the MSA, accurately reproducing high order statistics. They also show that generated sequences are relatively distant from those found in the MSA, showing the model is able to generalize.

I found the model and results very interesting and the paper relatively clear. However, I found that the authors remain a bit superficial in the analysis of their results, even in the SI. I also have doubts about whether the good performance of *Generalist* in reproducing statistics of sequences is due to it also fitting phylogenetic relations between sequences, which is something I believe a generative model should avoid (or at least it should be discussed).

I list below many comments going more in detail.

**Comments 1**

If found the paragraph about the mathematical aspect of *Generalist* a bit confusing.

as far as I understand, *πanl* is the probability to observe state *a* at position *l given* a state *zn* of the latent space. This is a general relation and is unrelated to the training data and the alignment, but this is not clear from the text. Why not use a more explicit notation such as *P*(*σa*,*l*∣*zn*)? Maybe this is a misunderstanding on my part.

The reviewer is correct in their understanding. We use $\pi_{anl}$ as a more concise notation for the schematic figure 1. We have added a line explaining that both notations are equivalent in the paragraph under Eq 1 defining the probability (lines 141-142).

I found the sentence "Sequences are modeled as arising from their own Gibbs-Boltzmann distribution" (l. 128) to be a bit confusing. Shouldn't it be something like "Given a state of the latent variables *z*, a sequence is distributed according to a Gibbs-Boltzmann distribution containing only fields". Of course, a state of the latent variables is attached to each sequence of the alignment.

Based on the reviewer’s suggestion, we have modified the explanation wording in the main text (lines 108-111).

One has to read the SI to understand how sequences are actually sampled. Given that the main point of the article is the statistical properties of sampled sequences, I think this could go into the main text.

Thanks for the suggestion, we have implemented this change (lines 151-155).

**2**

The authors use Potts models and Variational Auto-encoders as a comparison to *Generalist*. However, it seemed to me that Restricted Boltzmann Machines are a very natural comparison. The main difference between RBMs and *Generalist* is that in the latter, hidden units (or latent variables) are fixed to some predetermined states *zn*, one for each sequence of the original alignment. In both setups,

once the latent state is fixed, all positions in the sequence are chosen independently.
I was a bit surprised to not find any discussion of this connection with RBMs, and am curious to ask the authors what they think about it.

We thank the reviewer for the suggestion. Indeed, our model is related to RBMs where instead of allowing a distribution over hidden variables, we fix them to specific values. We have now added some discussion about restricted Boltzmann machines (lines 135-137).

**3**

A question about training: is the likelihood a convex function? Does it have a clear minimum?

Illustrating this, I ran a simple numerical experiment using the code and notebooks that the authors provided. I trained *generalist* on an alignment of 400 sequences of length 2 being either "AC" or "CA". The magnetizations are such that letters "A" and "C" are heavily favored over other amino-acids, and there is a very strong correlation between the two positions.

I used a latent space dimension *k* = 1. As far as I understand, *generalist* should be able to perfectly reproduce the statistics with parameters such as

fields *θ*1 = *θ*0 ⋅ [−1,1,0,...,0] for the first position and *θ*2 = *θ*0 ⋅ [1,−1,0,...,0]

"temperatures" *z* > 1 for the "AC" sequences and *z* < −1 for the "CA" sequences.
At sampling time, if *z* > 1 is chosen, the most likely sequence is "AC". If *z* < 1 is chosen, the most likely sequence is "CA".

Here were the results:

with the default settings, *generalist* did not converge to the above described state, but instead to a state with *θ*1 ≃ *θ*2 ≃ [−1, −1, 1, ... , 1] and *z* ≃ 2 for all sequences. Of course, this reproduces the magnetizations, but not the correlations, as the four states "AA", "AC", "CA", "CC" will have the same energy.

when I initialized the model with the fields *θ*1 = *θ*0 ⋅ [−1,1,0,...,0] and *θ*2 = *θ*0 ⋅ [1, −1, 0, ... , 0], training nicely converged to the solution proposed above.

I conclude from this that training highly depends on the initial state of parameters, and that the likelihood may poses several local maxima. However, I could see no mention of this in the article. Did I misunderstand something?

We sincerely thank the reviewer for raising this important issue which we clearly missed in our original manuscript. Indeed, like variational autoencoders, the likelihood in our model is nonconvex and therefore potentially has multiple local minima. Moreover, the inferred model parameters are also likely to be sensitive to initialization [10,11]. To alleviate the problem, we now train multiple models starting from different initializations and use for analysis the one with the highest likelihood. This approach, while not guaranteed to solve the issue of local versus global optimization, tackles the problem underscored by the reviewer. We have now made large scale changes to the manuscript and have completely redone our analysis by repeating the optimization procedure multiple times for all proteins considered.

I think the authors should comment a bit more on the training procedure: does it strongly depend on the initial conditions? does the optimal *k* vary from training to training? why are the *z* initialized to be positive values, whereas using negative values could increase the expressiveness of the model?

We thank the reviewer for raising this important point. To ensure that the optimal latent space dimension does not depend on the initialization and training, we now run our algorithm using multiple runs and use an average across multiple runs to determine optimal latent space dimension. Notably, the optimal dimension decided using this approach is quite similar to the one determined using a single run. Moreover, agreeing with the reviewer, we now allow random initializations that are both positive and negative. This has now been explained in the manuscript (lines 191-200).

**4**

The authors find optimal values for the dimension *K* of the latent space using a quite interesting method, avoiding overfitting. I have questions regarding the results.

The optimal *K* varies significantly for different families: from *K* = 10 for mTOR to *K* = 70 for DHFR. I would appreciate a comment of the authors on this variation. Was that expected from the diversity of sequences found in the different alignments? Looking at Figure 2, it seems that the least diverse alignments have the lowest *K*: this could easily be explored quantitatively.

The reviewer’s intuition is indeed correct~ To show how the optimal latent dimension depends on diversity within the MSA, in Reviewer Figure 2, we plot the average minimum Hamming distance within the natural sequences (x axis) and the optimal latent dimension K (y axis). While a more detailed analysis is needed, based on the 5 families studied here, it appears that modeling less diverse MSAs requires a lower latent dimension K.

Reviewer Figure 2. Optimal latent dimension determined by the GENERALIST model as a function of the overall diversity within the MSA, quantified as the average fractional Hamming distance between closest pairs of sequences.

How robust is the optimal value of *K*? For instance for BPT1, going from *K* = 42 to *K* = 50 completely changes the distribution of Hamming distances. How much do these values change, say, when using different initial conditions for the training? As a suggestion, maybe plotting the optimized quantity as a function of *K* would give the reader more intuition?

It would be interesting to see how the reproduction of 2nd and higher order statistics behaves as a function of *K*. Curiously, this is done for VAE in the SI, but not for *Generalist*.

We thank the reviewer for this suggestion. We have changed main text figure Figure 2 to illustrate the robustness and tunability of the optimal latent dimension. Specifically, we compute average of the metric that we use to infer the optimal K across 10 different fits of the model (corresponding to 10 different initializations of the parameters).

We have now included in the supplementary material (Supplementary Figure 7, Reviewer Figure 3), the effect of increasing the latent dimension K on the correlation metric $r_{20}.$ There is a direct relation between increasing the quality of capturing the statistics (higher $r_{20}$) and increase in the latent dimension since it increases the complexity of the model and allows the probabilities to fit to the data. However, as expected, this comes at a cost of overfitting to the data.

Reviewer Figure 3. The r20 metric plotted as a function of the dimension of the latent space. Higher dimensional latent spaces correspond to more complex models which in turn lead to a better fit to data albeit with a loss of generality.

**5**

In figure 5, the authors show that for large alignments, the minimal hamming distance between pairs of *Generalist* generated sequences and the minimal distance to a natural sequence is close to the ones found for the natural MSA and on average much lower than when using VAE and Potts models.

First, this result is not completely surprising as *K* has been optimized exactly for this. Seconds, I have the impression that this means *Generalist* reproduces the phylogenetic bias found in the natural sequences. If this is the case, the good performance of *Generalist* in reproducing high order statistics could be due to the fact that these statistics are indicative of phylogenetic relations and not of functional constraints. These are relations that Potts models try *not* to learn. I think it is important that the authors this potential pitfall.

We thank the reviewer for raising the very important point about phylogeny. We note that phylogenetic signature is always present in multiple sequence alignments. The reviewer correctly points out that other methods have tried to remove this signature with *ad hoc* corrections to the data. This is typically done by assigning weights to sequences based on sequence density and thereby lowering the importance of highly clustered sequences. We note that this method, though reasonable, may only correct for short term evolutionary history and does not correct for deep phylogenetic signatures [12]. We also note that the problem of removing the null expectation arising from phylogeny is a difficult one and no current methods fully solves it. Finally, this phylogenetic correction is independent of adabmDCA (Potts model) and ArDCA and can in principle be applied to any MSA-based algorithm. We have now addressed this in the manuscript (lines 212-214).

To provide a fair comparison, we do not include phylogenetic corrections for any of the models trained in this study, including GENERALIST, VAE, ArDCA, and adabmDCA. Moreover, we optimize for ArDCA and VAE hyperparameters to avoid overfitting the same way we do for GENERALIST.

**6**

The authors use Potts models as a comparison for *Generalist* and discuss them at length in the introduction. A major difference between Potts models (and also RBMs) and more involved techniques such as VAEs or neural networks is that the parameters of the former have a biological interpretation (to some extent). This is even pointed out in the introduction, as a downside of deep generative models (l. 90).

Did the authors explore possibilities to interpret the inferred parameters of *Generalist*?

We thank the reviewer for this excellent suggestion. Indeed, unlike other methods, the latent space embedding allows us to classify sequences with similar covariance structure, therefore, naturally identifying sectors in the multiple sequence alignment.

To show this, we used Gaussian mixture modeling (GMM) to cluster latent variable representations of sequences. GMMs imagine that the data (in our case, the latent space descriptors) arise from a mixture of several multivariate Gaussian variables. The parameters of these Gaussian distributions (mean value and covariances) as well as the membership of individual data points to the Gaussians can be determined using well-established algorithms such as the expectation-maximization algorithm. We use a built-in package in python to carry out these calculations.

The optimal number of clusters can in principle be determined using information theoretic criteria. However, given that the data are correlated with each other through an evolutionary process, we used the adjusted Rand index (ARI) which measures the reproducibility of the model [9]. Briefly, the cluster number N with the largest ARI corresponds to the most stable cluster assignment.

As shown in Reviewer Figure 4 (main text Figure 6), our findings suggest that the clusters generated by this process effectively encapsulate distinct statistical characteristics of the sequences in the training data. Our conclusions stem from an extensive analysis of these clusters, which involved computing the covariance and mutual information within each cluster. Briefly, our analysis identified 4 clusters in the data. The largest of these clusters comprised 73% of the total data and the other 3 clusters comprised 11%, 10%, and 6% of the data. respectively. Unsurprisingly, the largest cluster primarily preserved the statistical characteristics of the entire MSA. However, our investigation into the smaller clusters yielded a variety of results.

For example, as seen in Reviewer Figure 4, we discovered that each cluster conserved different pairs of amino acids, indicating the uniqueness of each cluster. Further, the mutual information between pairs of positions also varied from cluster to cluster. To ensure that these variations were not merely a result of differing cluster sizes, we constructed a null model. The null model involved random assignment of sequences to clusters that mirrored the size distribution of clusters inferred by GMM. Interestingly, these null clusters reproduced the original MSA's statistics much more closely than their GMM counterparts (SI Figure 5).

Reviewer Figure 4. Statistics of clusters from GMM for BPT1. Panel A. Comparison of covariance calculated from natural sequences within each cluster (labeled 0, 1, 2, and 3) and the entire MSA. B. Comparison of mutual information calculated between pairs of positions from natural sequences within each cluster and the entire MSA. Cluster sizes were 1774, 1034, 1623, and 12138 for Clusters 0, 1, 2, and 3 respectively.

**Minor comments**

In the introduction (around l.78), the authors make well-argued case against hyper-parameters used in training Potts models. Does *Generalist* use regularization? If not, how does one avoid inifinite values for some parameters?

We thank the reviewer for the question. For data analyzed in the manuscript, we stop our gradient descent algorithm when the gradient goes below a pre-specified threshold. This avoids runaway parameters. The code provided with the manuscript allows the user to set that threshold. Notably, GENERALIST uses fewer hyperparameters than other methods tested.

Following reviewer’s suggestion, we now allow the user to incorporate L2 norms on the latents and the features in the model. We have modified the log-likelihood cost function to reflect this L2 norm. We note that the analyses presented in the manuscript did not use any regularization.

The optimizer ADAM also has some hyperparameters: do those influence predictions (if the likelihood has a single minimum, I imagine they should not)? I think the article would benefit from a more explicit discussion of these issues.

We thank the reviewer for the comment. GENERALIST is in fact learnt with a reasonable speed for all tested MSAs and therefore did not require optimization of adam parameters. We therefore did not experiment with hyperparameters related to adam. However, given the nonconvexity of the problem, we do expect different initializations and adam parameters may reach different local maxima of the log likelihood. We now mention this in the manuscript (lines 191-200). Moreover, to make the inference robust to multiple local optimal solution, we train GENERALIST 10 times and choose the best.

In the paragraph starting at line 140, the authors describe the protein families used in the article. I am a bit annoyed by the use of the "approximate" sign ∼. Why not give the exact values of number of sequences and amino acid lengths? Also, a table would be really helpful here: it could contain number of sequences, length, optimal *K* and fitting quality for the different families.

We thank the reviewer for the suggestion. We have made appropriate changes (lines 157-163).

Code: it would be nice to provide installation instructions. Generalist depends on many other python libraries (torch, numpy, etc...), and I had to "manually" figure out which and install them.

Thanks for the suggestion, the required packages are in requirements.txt which has been moved to the highest-level folder in the repo.

l.73 - "inefficient" might not be the best word. Maybe "computationally hard"? l.308 - I am not sure that one can refer to Potts models as "physics-based".

Thanks for the suggestion, we have implemented the change.

**Reviewer #3**: In this manuscript, the authors propose a generative model, called GENERALIST, to model natural protein multiple sequence alignments (MSA). The model is very simple and this makes it extremely appealing. It consists in constructing K "profile models", interpreted as K features that describe the sequences. Each natural sequence is then represented as a linear superposition of these K profile models, with sequence-dependent weights Z.

Once the parameters are inferred, one can extract the weights Z corresponding to one of the natural sequences, and sample new sequences according to the profile models associated with it. Because of the profile structure, each aminoacid in the sequence is sampled independently, which makes sampling easy and efficient.

The number K of features is chosen in such a way as to match the probability distribution of the sequence identity with the closest natural sequence in the MSA for (a) sequences generated from the model and (b) natural sequences.

The authors then test their model on 5 different natural MSA, and compare it with other state-of-the-art approaches such as Boltzmann machines (adabmDCA), autoregressive models (arDCA), and variational autoencoders (VAE). They claim that GENERALIST outperform these models. The claim is based on a series of observations:

1) Higher-order statistics of the natural MSA is better reproduced (Fig.3)

2) Local maxima of the model probability are closer to natural sequences and AlphaFold2 predicts them to fold better into the natural structure associated to the family (Fig.4).

3) GENERALIST closely reproduces the distributions of distances between pairs in the generated vs natural ensemble, of minimum distances within the ensemble and with the natural MSA.

Given these facts, my assessment of the paper is the following. I think that the method is definitely of interest and as such I would recommend publication on PLOS computational biology. However, I am not fully convinced that GENERALIST outperforms existing models and I would thus encourage the authors to tune down some statements that I find a bit too bold.

The reason is the following. Models such as adabmDCA, arDCA and VAE assume that sequences in the natural MSA are identically and independently generated from an unknown probability distribution, which is learned by the inference method. On the contrary, GENERALIST tries to learn a model of the whole MSA. In doing so, GENERALIST incurs the risk of overfitting the phylogeny that is always present in natural MSAs; while the other methods try to get rid of it (perhaps without much success).

We thank the reviewer for this important comment. The reviewer is correct in noting that GENERALIST can run the risk of overfitting to the MSA. This will correspond to a model with a very high latent dimension. However, GENERALIST is also equipped with a straightforward mechanism to avoid overfitting which we use in our study to determine the optimal latent space dimension.

Specifically, when the latent space dimension is low, the generated sequences are significantly different from the natural sequences and the average distance between generated sequences and the closest natural sequence is high. When the latent space dimension is too high, the generated sequences are very close to the natural sequences and the distance between generated sequences and the closest natural sequence is low. However, we can use the average distance between nearest neighbor natural sequences as a metric to identify the correct latent space dimension. As shown in Reviewer Figure 5 (main text Figure 2), this metric allows us to pick an optimal latent dimension that can generate novel sequences while reproducing non-trivial sequence statistics. This way, we believe that GENERALIST avoids the risk of overfitting.

**Reviewer Figure 5.** **Optimization for the latent dimension for BPT1 (left panel) and EGFR (right panel).** The optimized value $\Delta^{2}=\left( \left\langle H_{min} \right\rangle_{\mathrm{generated}}- \left\langle H_{min} \right\rangle_{\mathrm{generated}} \right)^{2}$(y axis) is plotted against the latent dimension $K$ (x axis). For each sequence in an ensemble (natural or generated) $H_{min}$ is calculated by obtaining the minimum fractional Hamming distance to the natural sequences. Each box plot represents 10 runs for each latent dimension. The optimum latent dimension is determined as the one that minimizes the average value of $\Delta^{2}$ across the 10 runs. Optimal dimension for BPT1 is 42, and for EGFR is 19.

In this sense, it is not surprising that GENERALIST does a better job in fitting the distance relationships of the natural MSA and the high-order statistics. Methods such as adabmDCA, by construction, try to get rid of the low-distance tail that characterizes the natural MSA. I am therefore a bit afraid that GENERALIST might be overfitting the phylogeny a bit. Whether this is a problem or not depends probably on the task that one is considering, and therefore GENERALIST might well outperform the other approaches in some applications. But I would encourage the authors to be a bit more cautious in the absence of an experimental comparison of the methods on a given task that is relevant for biology.

We thank the reviewer for raising the very important point about phylogeny. We note that phylogenetic signature is always present in multiple sequence alignments. The reviewer correctly points out that other methods have tried to remove this signature with *ad hoc* corrections to the data. This is typically done by assigning weights to sequences based on sequence density and thereby lowering the importance of highly clustered sequences. We note that this method, though reasonable, may only correct for short term evolutionary history and does not correct for deep phylogenetic signatures [12]. We also note that the problem of removing the null expectation arising from phylogeny is a difficult one and no current methods fully solves it. Finally, this phylogenetic correction is independent of adabmDCA (Potts model) and ArDCA and can in principle be applied to any MSA-based algorithm. We have now addressed this in the manuscript (lines 212-214).

To provide a fair comparison, we do not include phylogenetic corrections for any of the models trained in this study, including GENERALIST, VAE, ArDCA, and adabmDCA. Moreover, we optimize for ArDCA and VAE hyperparameters to avoid overfitting the same way we do for GENERALIST.

A minor point: in the abstract and introduction, “density of sequences” is not clear. I guess it should be “probability density of sequence distances”.

We thank the reviewer for this suggestion. We have implemented this change in several places in the main text.

References

1.	Figliuzzi M, Barrat-Charlaix P, Weigt M. How Pairwise Coevolutionary Models Capture the Collective Residue Variability in Proteins? Molecular Biology and Evolution. 2018;35: 1018–1027. doi:10.1093/molbev/msy007

2.	Trinquier J, Uguzzoni G, Pagnani A, Zamponi F, Weigt M. Efficient generative modeling of protein sequences using simple autoregressive models. Nat Commun. 2021;12: 5800. doi:10.1038/s41467-021-25756-4

3.	Muntoni AP, Pagnani A, Weigt M, Zamponi F. adabmDCA: adaptive Boltzmann machine learning for biological sequences. BMC Bioinformatics. 2021;22: 528. doi:10.1186/s12859-021-04441-9

4.	McGee F, Hauri S, Novinger Q, Vucetic S, Levy RM, Carnevale V, et al. The generative capacity of probabilistic protein sequence models. Nat Commun. 2021;12: 6302. doi:10.1038/s41467-021-26529-9

5.	Amit DJ, Gutfreund H, Sompolinsky H. Storing Infinite Numbers of Patterns in a Spin-Glass Model of Neural Networks. Phys Rev Lett. 1985;55: 1530–1533. doi:10.1103/PhysRevLett.55.1530

6.	Mariani V, Biasini M, Barbato A, Schwede T. lDDT: a local superposition-free score for comparing protein structures and models using distance difference tests. Bioinformatics. 2013;29: 2722–2728. doi:10.1093/bioinformatics/btt473

7.	Jumper J, Evans R, Pritzel A, Green T, Figurnov M, Ronneberger O, et al. Highly accurate protein structure prediction with AlphaFold. Nature. 2021;596: 583–589. doi:10.1038/s41586-021-03819-2

8.	David A, Islam S, Tankhilevich E, Sternberg MJE. The AlphaFold Database of Protein Structures: A Biologist’s Guide. Journal of Molecular Biology. 2022;434: 167336. doi:10.1016/j.jmb.2021.167336

9.	Rand WM. Objective Criteria for the Evaluation of Clustering Methods. Journal of the American Statistical Association. 1971;66: 846–850. doi:10.1080/01621459.1971.10482356

10.	Dixit PD. Thermodynamic inference of data manifolds. Phys Rev Research. 2020;2: 023201. doi:10.1103/PhysRevResearch.2.023201

11.	Zhao X, Plata G, Dixit PD. SiGMoiD: A super-statistical generative model for binary data. Welch J, editor. PLoS Comput Biol. 2021;17: e1009275. doi:10.1371/journal.pcbi.1009275

12.	Qin C, Colwell LJ. Power law tails in phylogenetic systems. Proc Natl Acad Sci USA. 2018;115: 690–695. doi:10.1073/pnas.1711913115
