# Supplementary material for: GENERALIST: A latent space based generative model for protein sequence families
Source: PLoS Comput Biol. 2023 Nov 27;19(11):e1011655. doi: 10.1371/journal.pcbi.1011655 (PMC10703406; doi:10.1371/journal.pcbi.1011655)
Supplement: Attachment — Submitted filename: Response_to_reviewers.docx [file pcbi.1011655.s015.docx]

Reviewer #1: I thank the reviewers for their responses, which have improved the paper. However, I must push back on a few points where the responses are inaccurate. In addition, one finding from the response raises some concerns.

1. In response to Reviewer 1 Comment 1: “However, while these approximate methods can potentially predict the effect of mutations and physical contacts between residues, they perform poorly when it comes to reproducing statistics of real sequences, which was the chief goal of our generative models and also of our work. The inaccuracy of approximate inference methods (pseudolikelihood, mean field, adaptive cluster expansion, etc.) in reproducing statistics of natural sequences was extensively explored by Figliuzzi et al. [1].”

The statement on the inaccuracy of alternative inference methods is not correct, as the authors can verify by consulting this reference (Figliuzzi et al., MBE 2018). In Figliuzzi et al., it is shown that pseudolikelihood does not do a good job of reproducing the statistics of the data. It is well-known that mean field approaches also do not reproduce statistics of the data. However, Boltzmann machine learning works very ­­­­­­­well (Figliuzzi et al., MBE 2018). Similarly, (Barton et al., Bioinformatics 2016) show good reproduction of statistics of the data using the adaptive cluster expansion method. The same is also true for ArDCA and minimum probability flow.

We apologize for our mischaracterization. We have now corrected our claims in the manuscript. As the reviewer suggest, we now motivate our method using its own merits rather than a comparison with previous works (lines 102-112).

Continuing on, “We use the term “Potts models” to refer to a generalization of spin-glass models for categorical data wherein the “Hamiltonian” of any sequence is given by [Eq. R1]. […] We note that in our manuscript, we do not train Potts models for proteins with L > 2000. In fact, we only train the Potts model for BPT1 with L ~ 60 residues. What the reviewer may be referring to is our implementation of a previously developed autoregressive model (ArDCA) [2] which has a very similar probability distribution as the Potts model yet cannot be represented using a Hamiltonian in Eq. R1.”

This is splitting hairs, and by this same standard the model (or, if we are being very precise, the ensemble of models) inferred by pseudolikelihood is also not a Potts model.

We apologize for the confusion. We referred to Potts models as those whose generative likelihood was Ising-like. The generative likelihood of the ArDCA model contains unidirectional coupling parameters (as noted by the authors of that model themselves) and was therefore deemed to be not Ising-like. However, this distinction is not important for the central message of the manuscript, and we have therefore amended our discussion of ArDCA (lines 71-87).

In response to the number of hyperparameters used, the response to reviewers states that “We do not include the dimension of the latent space as a hyperparameter since its value can be computed from the data.” However, in the paper the authors state “The latent space dimension is tuned to achieve a user-desired tradeoff between the novelty of
generated sequences and the accuracy of the ensemble in reproducing properties of the natural MSAs.” (lines 112-115). So, the dimension is not a hyperparameter, but the cutoff used to determine the dimension is. One can compare GENERALIST with alternatives such as ArDCA and find a very similar number of hyperparameters.

We have now corrected our claim. Specifically, the number of hyperparameters in GENERALIST are quite similar to ArDCA but smaller than adabmDCA.

Broadly, I have two recommendations. The first, specific recommendation would be to ensure that claims about previously developed methods in the literature are accurate. Secondly, I would suggest that the authors either motivate their work on its own merits – and indeed, their approach is interesting on its own – or substantiate claims about performance with data in this paper.

We thank the reviewer for the comments, and we accept the criticism. We have now motivated our work based on its own merits, specifically, as an easily implementable, accurate and interpretable alternative to currently available methods (lines 102-112).

2. In response to Reviewer 1 Comment 4 and related comments from Reviewers 2 and 3, “Parameter inference in GENERALIST is indeed nonconvex with potential to get stuck in local optima. Based on the comments of this reviewer and others, we now alleviate this issue by training the GENERALIST model multiple times and choosing the one that has the highest likelihood. The latent factors are not similar between different runs, due to a variety of previously documented indeterminacies [10,11].”

This finding seems concerning for the interpretation of the model, if not its fit to the data. Are other features of the model, such as local sequence optima or sequence clusters, conserved between different runs? Of course, if a particular parameter initialization turns out to be a bad one and the fit is poor, then such an instance could be discarded. Here, I am asking whether or not similarly good fits to the data return similar insights.

The authors should note whether or not these findings are consistent, and if not, this point should at least be acknowledged and explained in the Discussion.

We thank the reviewer for raising this important issue. Based on the reviewer’s suggestion, we checked the stability of the obtained clusters. Specifically, we ran our inference 10 times twice. In each of these 10 runs, we selected the model with the best likelihood and performed clustering analysis on the two independent models. As shown in reviewer Figure 1, the mixture Gaussian clustering robustly identified two clusters that were reproducible across the two runs. Notably, the statistics of the sequences were significantly different in the two clusters. We have now included this result in the text (lines 350-364, Figure 6).

Reviewer Figure 1. Jaccard Index for different number of clusters in the GMM.

Reviewer #2: I thank the authors for their work on revising the manuscript. My comments have been addressed.

Reviewer #3: I read with great interest the authors' answers to the referees. Both the questions and the answers were very insightful. I confirm my previous assessment of the paper and suggest acceptance on PLOS Computational Biology.

However, I would like the authors to consider the following comments, based on their answers to the other referees. Lines below refer to the authors' answers to the referees:

1. (Lines 125-126) The authors have removed claims of computational efficiency. Why? I would suggest adding a table with the training and sampling times for the various models, which will be very useful for future reference.

We thank the reviewer for the comment. We have not added these comparisons (and removed them from our previous version) because we realized that have not performed a systematic analysis. For example, these calculations were performed on different machines with different specifications (some on GPUs others on CPUs), making the comparisons scientifically invalid. Therefore, we think that it is most parsimonious to not make any claims about training and sampling times.

2. (Lines 181-182) The authors mention the presence of spurious optima in bmDCA. I am not totally sure that the presence of local optima that are distinct from the natural sequences is a bad sign. On the contrary, it could be a sign that bmDCA is able to generalize. The only way to know this is to perform experimental tests of these sequences.

We thank the reviewer for the comment. We agree that presence of local optima distinct from natural sequences indeed is potentially encouraging. However, the sequence features of the optimal sequences suggest that these are artificial sequences with little probability of folding into stable structures. For example, the amino acid composition appears to be unnatural: as seen in Reviewer Figure 2, the composition of these sequences is significantly different from natural sequences. While not conclusive, this suggests that the inferred optimal sequences are likely to not represent foldable sequences.

Reviewer Figure 2. Amino acid usage frequency in natural sequences (blue) and inferred optimal sequences (green). The amino acids are arranged according to their frequencies in natural sequences (from highest to lowest).

3. (Lines 222-229) The fact that GENERALIST does not account for epistasis in important. It should be mentioned explicitly in the main text.

We have now incorporated this in the manuscript (lines 143-149).

4. (Lines 436-437) Are the authors sure that repeating the training 10 times is enough to solve the problem? (Did they check it in the specific example proposed by referee 2?)

We further investigated the Reviewer 2 example to test this. We show below that 10 runs are likely to be sufficient to overcome the problem of local optima. To show this, we show a plot of the log likelihood curves for different runs, starting from different initializations. As we can see, while some runs get stuck a shallow local optima, multiple runs do converge to a deeper minimum with the highest log likelihood.

Reviewer Figure 3. Log likelihood of the toy model as a function of number of iterations.

What would be a good criterion to decide how many times the training should be repeated?

We thank the reviewer for this excellent question. Given the complex nature of the optimization, we do not believe that we can have good analytical understanding of the number of training repetitions. However, based on our models of several proteins suggests that performing the calculations ~10 times already results in several fits that preserve the generative performance (lines 195-200). We have now clarified this point in the supplementary material (SI lines 38-43).

5. (Lines 407-409) When talking about RBMs, it should be noted that RBMs have been applied to protein families in a series of works mostly by the group of Cocco and Monasson, e.g. "Learning protein constitutive motifs from sequence data", eLife, <https://doi.org/10.7554/eLife.39397.001>

We have now included this reference in our manuscript (line 131).

A more direct comparison with these approaches, most notably comparing the latent space representations, would be nice, but I understand that it goes beyond the scope of this work. However, perhaps some of these works could be cited. (I wish to make clear that I am not an author in any of these papers, so there is no conflict of interest here.)

6. On the question of phylogeny, which was my most important concern and was shared by the other referees, I am not totally satisfied by the answer. I remain of the opinion that the other approaches work under different assumptions about phylogeny, such that a direct comparison might be misleading. However, I agree with the authors that this is one of the most important issues in generative modeling of proteins, and I think that GENERALIST will contribute a new tool to tackle it, so I accept the authors' point of view on this and do not require any change to the paper.

We thank the reviewer for the comment. We are actively exploring how to incorporate effects of phylogeny in GENERALIST. We leave it to future studies.
